# Função Diastólica e Biomarcadores de Participantes de Caminhada de Longa Distância

**DOI:** 10.36660/abc.20190271

**Published:** 2020-10-13

**Authors:** Maicon Borges Euzebio, Priscila Valverde de O. Vitorino, Watila Moura Sousa, Milena Andrade Melo, Sérgio Henrique Nascente Costa, Ana Luiza Lima Sousa, Thiago de Souza Veiga Jardim, Ana Carolina Arantes, Paulo Cesar B. Veiga Jardim, Weimar Kunz Sebba Barroso

**Affiliations:** 1 Universidade Federal de Goiás GoiâniaGO Brasil Universidade Federal de Goiás – Medicina, Goiânia, GO – Brasil; 2 Pontifícia Universidade Católica de Goiás GoiâniaGO Brasil Pontifícia Universidade Católica de Goiás, Goiânia, GO – Brasil; 3 Universidade Federal de Goiás GoiâniaGO Brasil Universidade Federal de Goiás – Pós-graduação em Ciências da Saúde, Goiânia, GO – Brasil; 4 Universidade Federal de Goiás GoiâniaGO Brasil Universidade Federal de Goiás – Liga de Hipertensão Arterial, Goiânia, GO – Brasil; 5 Universidade Federal de Goiás GoiâniaGO Brasil Universidade Federal de Goiás – Cardiologia, Goiânia, GO – Brasil; 6 Faculdade da Polícia Militar do Estado de Goiás GoiâniaGO Brasil Faculdade da Polícia Militar do Estado de Goiás, Goiânia, GO - Brasil

**Keywords:** Caminhada, Biomarcadores Biológicos, Pressão Sanguínea, Troponina T, Peptídeo Natriurético Cerebral, Atletas, Ecocardiografia Doppler/métodos

## Abstract

**Fundamento:**

Os efeitos da caminhada de longa distância sobre o sistema cardiovascular são pouco estudados.

**Objetivos:**

O objetivo geral deste estudo foi verificar esses efeitos sobre o comportamento da função diastólica e dos biomarcadores cardíacos CK-MB (massa), troponina T e NT-proBNP em atletas amadores.

**Método:**

Este estudo longitudinal realizado em 2015 avaliou os participantes nas 5 etapas seguintes: A0 (basal) antes de iniciar o percurso, e as demais, A1, A2, A3 e A4 ao final de cada dia, totalizando 244,7 km. Em todas as etapas foram dosados os biomarcadores NT-proBNP, CK-MB (massa) e troponina T. Realizou-se ecocardiograma para análise das ondas E, A e E'. Adotado p < 0,05 como significativo.

**Resultados:**

Foram avaliados 25 participantes com média de idade de 46 ± 10,5 anos e índice de massa corporal de 20,2 ± 2,3 kg/m2. Encontrou-se aumentos dos valores de NT-proBNP de A0 para A1, A2, A3 e A4 (p < 0,001), CK-MB (massa) de A0 para A2 (p < 0,001) e da onda E' de A0 para A1, A2, A3 e A4 (p < 0,001). Foram identificadas correlações entre os seguintes: CK-MB (massa) e troponina T (A1: r = 0,524, p = 0,010; A4: r = 0,413, p = 0,044); CK-MB (massa) e NT-proBNP (A4: r = 0,539, p = 0,006); e E/A e E' (A0: r = 0,603, p < 0,001; A1: r = 0,639, p < 0,001; A4: r = 0,593, p = 0,002); e correlação negativa entre CK-MB (massa) com E/A (A1: r = −0,428, p = 0,041).

**Conclusão:**

Os efeitos da atividade física intensa, prolongada e intercalada foram verificados a partir das variações significativas no comportamento da CK-MB (massa), NT-proBNP e E'. Apesar das alterações encontradas, não houve critérios sugestivos de dano ao miocárdio.

## Introdução

O exercício físico é fundamental para a manutenção da saúde geral e prevenção de doenças.^1^ Entretanto, a intensidade, duração e frequência são fatores que separam os benefícios dos prejuízos no organismo humano. Evidências recentes questionam se a demanda fisiológica para manter um débito cardíaco elevado durante um período prolongado de exercício pode resultar em comprometimento transitório das funções cardíacas.^2^

Os efeitos agudos do exercício físico na função diastólica e até mesmo as variações de disfunção diastólica estão relacionadas indiretamente à estimulação persistente, dependendo também da intensidade e duração. Essas adaptações, mesmo em curto prazo, podem produzir diminuição da função cardíaca, fato esse conhecido como fadiga cardíaca.^3^

As enzimas cardíacas creatina quinase fração MB (CK-MB, *creatine kinase-MB*), troponina T cardíaca e do aminoácido precursor N-terminal do peptídeo natriurético cerebral tipo B (NT-proBNP, *N-terminal pro B-type natriuretic peptide*) são biomarcadores importantes para a avaliação da existência de lesões miocárdicas e da função diastólica.^4^

Níveis de CK-MB também podem elevar em estados de rabdomiólise e até de apoplexia, possuindo dessa forma uma sensibilidade variável.^5^ A troponina T é o marcador preferencial de lesões miocárdicas, considerado como padrão ouro.^6^

O NT-proBNP está associado direta e paralelamente com as concentrações do peptídeo natriurético cerebral (BNP – *brain natriuretic peptide*). Ele pode ser utilizado para avaliação diagnóstica e prognóstica da insuficiência ventricular esquerda e também se eleva em condições que induzem disfunção diastólica. Pouco se conhece sobre o comportamento desses biomarcadores e especialmente do significado prognóstico em indivíduos saudáveis submetidos a stress físico intenso.^7^

No entanto, poucas pesquisas avaliaram o efeito da caminhada de longa distância, de moderada a alta intensidade, no sistema cardiovascular a partir da avaliação da função diastólica e dos biomarcadores cardíacos. Este foi o primeiro estudo a avaliar a função diastólica e os biomarcadores cardíacos nesse tipo de exercício. Dessa forma o objetivo do presente estudo foi verificar, em atletas amadores, os efeitos de caminhada de longa distância, de moderada a alta intensidade, sobre o comportamento da função diastólica e dos biomarcadores cardíacos CK-MB (massa), troponina T e NT-proBNP.

## Métodos

Este foi um estudo longitudinal, realizado durante caminhada de longa distância no ano de 2015 - 24ª Caminhada Ecológica de Goiás. Inicialmente, cerca de duzentas pessoas se inscreveram pela internet para participar. Os candidatos participaram de uma seletiva, na qual percorreram 56 km, divididos em dois dias (28 km cada), os quais deveriam ser cumpridos em até três horas e dez minutos para os homens e três horas e 30 minutos para as mulheres. Os classificados de acordo com o melhor tempo de cada faixa etária foram selecionados, totalizando 29 participantes, sendo 25 do sexo masculino e quatro do sexo feminino. As mulheres foram retiradas da análise por não completarem diariamente o percurso previamente estabelecido.

O projeto de pesquisa foi aprovado pelo Comitê de Ética em Pesquisa da Pontifícia Universidade Católica de Goiás sob parecer de número 1.107.021. Os indivíduos aprovados na seletiva foram convidados a participar do estudo. Após o aceite, aplicou-se o Termo de Consentimento Livre e Esclarecido e foi realizada a avaliação inicial (A0) em uma unidade de avaliação cardiovascular. As outras avaliações aconteceram 34 dias após A0 ao longo do trajeto de 244,7 km, ao final de cada um dos dias e nos locais de repouso de cada cidade do percurso no período de 21 a 24 de julho de 2015 sendo cognominadas de A1, A2, A3 e A4 (avaliação do 1º dia 2º dia 3º dia e 4º dia). Não foram realizadas coletas de dados no último dia do evento pois os participantes foram dispensados para retornarem às suas cidades.

A dominância do relevo foi dada em subida, em descida e nivelado, conforme cada dia do percurso. Essas informações foram obtidas de um estudo anterior que já havia avaliado esse percurso.^8^ A declividade do traçado do terreno foi obtida por meio do *software* *ArcGIS 10.3* pela ferramenta de declividade (*slope, arctoolbox*), a partir das imagens *Shuttle Radar Topography Mission* (*SRTM*) fornecidas pelo serviço geológico americano de três arcseg de tamanho de pixel. Para a monitorização meteorológica dos dias do percurso (A1 a A4) foram utilizados os dados da estação Goiás denominada oficialmente pelo Instituto Nacional de Meteorologia - INMET de A014.

### Organização da Coleta

O ambiente de pesquisa foi subdividido em estações, uma para cada avaliação. As avaliações foram compostas por anamnese (somente em A0); ecocardiografia e coleta de sangue para avaliação de biomarcadores em todas as etapas. As coletas dos dados ecocardiográficos e biomarcadores começavam por volta das 18 horas (30 a 120 minutos após o final da caminhada) de cada dia do percurso. Dessa forma, quando os participantes chegavam nos locais de repouso, já haviam sido preparadas estações de pesquisa iniciando o fluxo pela identificação dos participantes e depois pela coleta de sangue e avaliação ecocardiográfica, sendo posteriormente dispensados para voltarem a rotina do evento. Não foi possível realizar coleta pela manhã pois os participantes levantavam às 04 horas da madrugada e isso poderia prejudicá-los por interferir nos seus períodos de descanso e recuperação.

A anamnese foi organizada pelos pesquisadores em formato de entrevista com questões referentes a dados pessoais como idade (em anos), sexo (masculino e feminino), profissão e estado civil; antecedentes pessoais e familiares: hipertensão arterial, diabetes e dislipidemia; uso de medicamento(s); tabagismo (atual/anterior); quantidade de cigarros por dia para fumantes; prática de exercício físico (frequência semanal, duração diária e tempo de prática); presença de problema ortopédico que dificultasse a realização de exercícios.

### Procedimentos para a Realização dos Exames de Biomarcadores Sanguíneos e Ecocardiograma

Foi utilizada a técnica para a coleta de sangue por meio de acesso venoso periférico em fossa cubital esquerda.^9^ Foram extraídos cerca de 5ml de sangue de cada participante. O sangue foi centrifugado no local da coleta a 3.000 rpm durante 10 minutos para a obtenção do soro/plasma. O soro foi colhido em tubos de amostra com gel separador. As amostras foram prontamente armazenadas e congeladas a −20 ºC em congelador específico nas estações de coleta.^10^ As amostras foram encaminhadas para um laboratório credenciado para serem analisadas. Para a realização dos exames dos biomarcadores foram utilizados kits com registro na Agência Nacional de Vigilância Sanitária (ANVISA). Os valores de CK-MB (massa), troponina T e NT-proBNP foram obtidos a partir da técnica imunoensaio de electroquimioluminescência.^11^ Os ensaios foram realizados pelo sistema COBAS® - Modular Analytics E170 a partir dos kits seguintes: CK-MB STAT, Troponin T hs e proBNP II respectivamente. Para assegurar a correta execução do ensaio, foram cumpridas todas as instruções fornecidas no documento guia para o analisador. Todos os exames foram processados por biomédico. Foram utilizadas as seguintes unidades de medidas: pg/ml para NT-proBNP e para troponina T e ng/ml para CK- MB (massa).^11^

### Ecocardiografia

O ecocardiograma foi realizado individualmente em todas as etapas da coleta. Em A0 ao longo do dia e, de A1 até A4 por volta das 18 horas, após a chegada dos participantes nas estações de pesquisa. Todos os exames foram realizados pelo mesmo ecocardiografista. Foi realizado ecocardiograma uni e bidimensional com Doppler colorido, utilizando-se o aparelho portátil Philips CX50®, com transdutor eletrônico de 2-5MHz de frequência. A técnica e os padrões de referência utilizados foram aqueles preconizados pela *American Society of Echocardiography.*^12^ Foi utilizado o mesmo ângulo de incidência para análise transmitral, procurando-se alinhar o feixe de ultrassom o mais paralelo possível ao fluxo do Doppler colorido. Os parâmetros utilizados para a análise da disfunção diastólica, com suas respectivas unidades de medidas, foram, para fluxo mitral, E em cm/s, A em cm/s e a relação E/A. Para avaliação tecidual foi E' em cm/s.^13^

### Análise Estatística

Os dados foram analisados por estatística descritiva com frequências absolutas e relativas, médias, desvio padrão e intervalo de confiança. Para testar a normalidade da distribuição dos dados das variáveis foi utilizado o teste de Shapiro-Wilk. Para as comparações das variáveis referentes aos biomarcadores cardíacos entre os dias da caminhada e referentes aos parâmetros ecocardiográficos, foi realizado o teste de ANOVA para medidas repetidas, seguido do *post hoc* de Bonferroni. Para as correlações foram utilizados os testes de Pearson ou Spearman. Adotou-se o valor de p < 0,05 para significância estatística. Para realizar a análise estatística foi utilizado o software Stata, versão 14.

## Resultados

### Características da Amostra

Foram avaliados 25 homens, média de idade de 46 ± 10,5 anos e índice de massa corporal de 20,2 ± 2,3 kg/m^2^ (Tabela 1). Quatro (16%) participantes utilizavam medicamentos que não influenciaram nas variáveis analisadas. A distância percorrida em quatro dias foi de 244,7 km e velocidade média de 7,6 km/h. Todos os participantes caminhavam juntos, em bloco, mantendo portanto a mesma velocidade. O período de descanso noturno diário foi entre seis e sete horas.

**Tabela 1:** Características sociais, de saúde e de hábitos de vida dos participantes da Caminhada Ecológica de Goiás, Brasil, 2015, n = 25*

Variáveis	n	%
**Faixa etária (anos)**		
19 – 39	5	20
40 – 59	19	76
≥ 60	1	4
**Uso de medicamentos**		
Não	21	84
Sim	4	16
**Doenças relatadas**		
Diabetes mellitus	1	4
Dislipidemia	1	4
Hipotireoidismo	2	4
Problemas ortopédicos	3	12

* Número de participantes avaliados

Em relação aos exercícios físicos prévios, 24 participantes (96%) praticavam corrida; um (4%) ciclismo e quatro (16%) musculação. A média de tempo de realização prévia de atividade aeróbia foi de 13,3 anos (0,5 ± 40) e a média da distância semanal de corrida, que foi a atividade relatada como mais praticada, foi de 64 km.

### Características Ambientais do Trajeto

A temperatura durante os dias do percurso variou de 19 a 32 ºC e a umidade relativa de 21 a 77%. Em A1 e A3 o relevo era predominantemente de subida, em A2 de descida (A2) e em A4 plano. As variações mais acentuadas da declividade do terreno foram encontradas nos trechos de A2 e A3, variando de 0 a −15.

### Avaliação dos Biomarcadores e das Ondas de Função Diastólica Durante a Caminhada de Longa Distância

**Figura 1:**
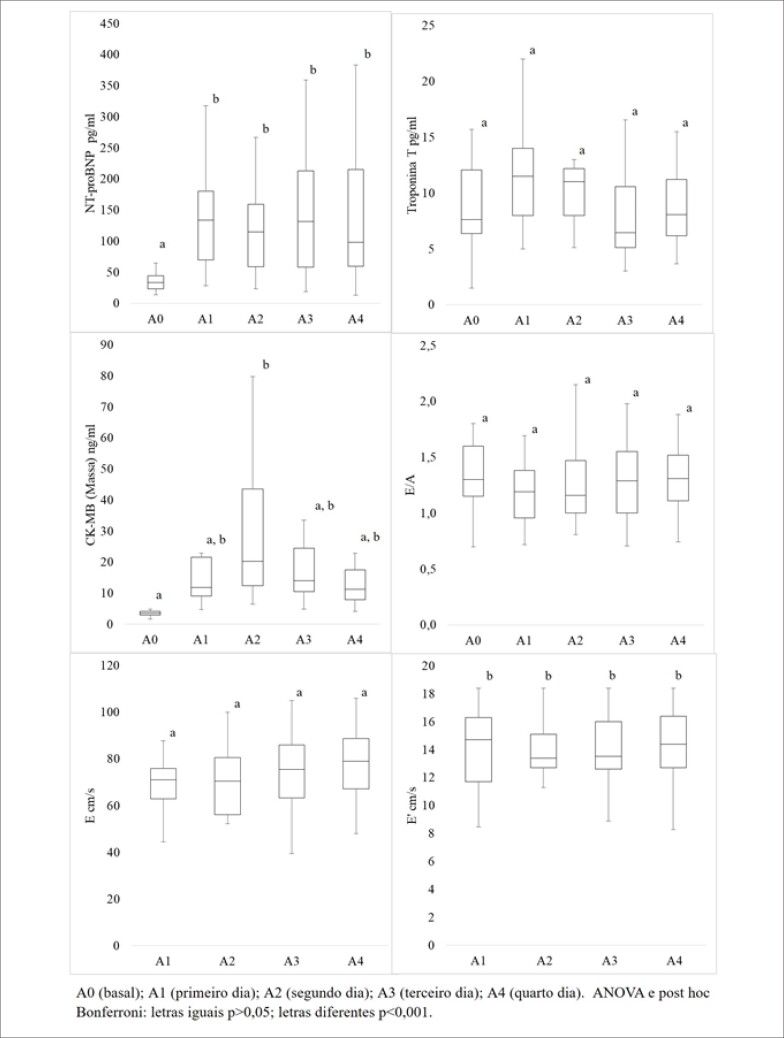
Comparação entre valores de NT-proBNP, Troponina T, CK-MB (Massa), E/A, E e E' obtidos durante a avaliação basal e durante os quatro dias de exposição à atividade física de moderada a intensa.

O NT-proBNP apresentou aumento significativo de A0 para todas as outras avaliações. A CK-MB (massa) teve aumento significativo de A0 para A2 e a troponina T não apresentou alterações significativas. Quanto a função diastólica, as ondas E e A não sofreram alterações entre os dias da caminhada. A onda E' aumentou de A0 para todos os outros dias (Figura 1).

Foram realizadas correlações entre todos os parâmetros coletados referentes aos biomarcadores e ondas da função diastólica e, evidenciadas somente as correlações significativas. Com relação aos biomarcadores sanguíneos, foram identificadas correlações positivas e moderadas nos seguintes dias de exposição: A1, entre troponina T e CK-MB (massa); A4, entre NT-proBNP e CK-MB (massa) e entre troponina T e CK-MB (massa). Na função diastólica, foram identificadas correlações positivas e moderadas entre E/A e E' em A0, A1 e A4. Dentre os biomarcadores e as ondas da função diastólica, foi evidenciada correlação negativa e moderada entre CK-MB e E/A em A1 (Tabela 2).

**Tabela 2:** Correlação dos biomarcadores, da função diastólica e dos biomarcadores com a função diastólica de participantes da Caminhada Ecológica de Goiás, Brasil, 2015, n = 25

Avaliação	Parâmetro	r	p
A0	E/A x E’	0,603	0,001*
			
	Troponina T x CK-MB (massa)	0,524	0,010**
A1	E/A x E’	0,639	0,001*
	CK-MB (massa) - E/A (seria “x”?)	-0,428	0,041**
			
	NT-proBNP x CK-MB (massa)	0,539	0,006**
A4	Troponina T x CK-MB (massa)	0,413	0,044**
	E/A x E’	0,593	0,001*

* Teste de Pearson;

** Teste de Spearman.

## Discussão

Apesar dos participantes serem considerados atletas amadores, a maioria possuía experiência prévia no percurso e em edições anteriores da Caminhada Ecológica de Goiás com uma média de 7,2 participações; somando-se a isso, o processo seletivo para ingresso proporcionou o surgimento de uma amostra altamente selecionada.

A velocidade de corrida competitiva em meia maratona ou maratona solicita maiores demandas energéticas e metabólicas e amplia a chances de desfechos cardiovasculares, principalmente em indivíduos não condicionados.^14^ No nosso trabalho a velocidade média foi de 7,6 km/h, bem inferior ao modelo de maratona e meia maratona e, portanto, com menor demanda energética e metabólica dos participantes.

Durante os dias de exposição ao esforço, em comparação aos 30 dias que precederam (A0), ocorreram aumentos significativos dos valores de NT-proBNP, CK-MB (massa) e da onda E' (Doppler tecidual). Não observamos variações significativas nos níveis de troponina T e na relação das ondas de fluxo mitral E/A.

Este aumento também foi verificado em indivíduos saudáveis, adolescentes e adultos, independente do sexo, quando submetidos a exercícios de resistência com retorno aos valores basais nas primeiras 24 horas após o exercício.^15^^,^^16^

No presente estudo, encontramos um aumento nos valores das concentrações séricas de NT-proBNP em até cinco vezes após o exercício quando comparado aos níveis basais, dados que corroboram com a literatura.^17^^–^^19^ Esses dados reforçam a importância do efeito natriurético no mecanismo de adaptação aguda e subaguda do aparelho cardiovascular ao esforço físico.

Elevações séricas da CK-MB (massa) também foram encontradas em indivíduos após exercícios extenuantes. Em uma pesquisa, realizada nos Estados Unidos com participantes jovens de uma maratona, foram encontrados níveis plasmáticos aumentados e sustentados da CK-MB em até 54 horas pós exposição ao exercício.^20^^,^^21^

Indivíduos treinados, que realizam exercício de alta intensidade em práticas esportivas, tendem durante o teste de esforço máximo apresentar de forma aguda maiores elevações séricas da CK-MB do que indivíduos não treinados, sugerindo assim um envolvimento cardíaco e principalmente benigno na natureza desses níveis séricos.^22^

Cientistas também verificaram o efeito do exercício prolongado e das variações de temperatura ambiental no comportamento das creatina quinases e identificaram que quanto mais prolongado o exercício e maior a temperatura ambiental, maiores são as liberações plasmáticas desses biomarcadores.^23^ Quanto mais extenso o trecho percorrido em uma menor quantidade de tempo, maiores as chances de lesões musculares e, portanto, a presença sérica de maior quantidade das creatina quinases. Mais um fato importante é que a força de reação do relevo, nos momentos descida, exercida contra os participantes, pode também ocasionar aumento de lesões musculoesqueléticas, devido à maior fonte de impacto com tendência a um custo metabólico mais acentuado durante o declive.^24^^,^^25^

As variações significativas da CK-MB (massa) em nosso estudo foram encontradas somente entre os trechos de A0 e A2. Este aumento pode ter sido relacionado às características do percurso em A2, a saber, predominantemente de descida, com maior variação de declive e com maior extensão quando comparado aos outros dias. O efeito cumulativo da CK-MB (massa) em A1 pode ter influenciado o aumento significativo do valor da CK-MB (massa) em A2.

Existem também comprovações das limitações da avaliação da CK-MB em indivíduos saudáveis durante a prática de exercício físico. Sua especificidade fica prejudicada na presença de processos inflamatórios e de estresse muscular esquelético associado à redução nas primeiras horas de exposição ao esforço pelo aparecimento lentificado desses marcadores no sangue, sendo mais sensível quando há presença de injúria cardíaca.^26^

Em estudos de meia maratona, maratona e ultramaratona de 48 horas, realizados em corredores amadores (indivíduos não atletas), foram encontrados valores elevados de troponina T nas três primeiras horas da pós-exposição com reduções importantes logo a seguir, chegando a níveis basais em no máximo 48 horas.^20^^,^^27^ Em nadadores, após 60 minutos de natação, também foi verificado a mesma variação da troponina T que em corredores amadores.^16^

Eijsvogels et al.^28^ avaliaram 82 pessoas durante um trajeto de 30 quilometros/dia durante quatros dias, também identificou que as troponinas tiveram aumento somente no primeiro dia com redução plasmática em todos os outros dias apresentando associação com a velocidade da caminhada.^28^ Uma meta-análise de 45 estudos que avaliaram o comportamento das troponinas e dos BNPs após a exposição a exercícios de resistência observou que os valores plasmáticos elevados das troponinas e BNP durante e após exercício intenso e prolongado são propensos a alterações, podendo representar uma característica aguda frente a exposição ao exercício.^29^ Ainda não está clara a biomecânica da liberação das troponinas induzida pelo exercício físico e se essa realmente reflete um processo fisiológico ou patológico.^30^

A troponina T avaliada no presente estudo não apresentou, durante os dias do percurso, aumento significativo e difere dos resultados encontrados em outros estudos citados. Isto pode estar relacionado aos fatos de os participantes terem percorrido os trajetos em uma velocidade média abaixo dos valores de competição mesmo para corredores amadores; terem alternado corrida com caminhada; e terem sido hidratados rigorosamente durante todo o trajeto. O comportamento da troponina T foi diferente do geralmente esperado quando relacionado a CK-MB (massa). Como a troponina T tem maior especificidade para dano miocárdico por isquemia é possível que essa seja a razão de menor aumento entre A0 e os demais dias. Este fato pode demonstrar que os danos ao sistema cardiovascular parecem ser mínimos e que essa modalidade de esforço aparenta ser segura.^31^

Em nossa amostra, encontramos aumento significativo da onda E' com relação aos valores basais e sem redução da relação das ondas E/A. Esses achados podem estar relacionados às características da população avaliada que era bem condicionada e à intensidade do esforço, concorrendo para uma maior capacidade adaptativa de remodelamento do miocárdio. Outros estudos que fizeram ecocardiografia em diferentes populações tiveram resultados distintos. Não foi encontrada disfunção diastólica ventricular esquerda ou direita em triatletas amadores de média e longa distância.^32^ Já em atletas adultos foram demonstradas alterações do relaxamento miocárdico durante a diástole,^33^ identificada pelo aumento da onda E (fluxo mitral) em comparação com a onda E' (Doppler tecidual). Em exercícios de ultra-resistência houve diminuições significativas na onda E e na relação E/A imediatamente após o exercício.^34^^–^^36^ Uma publicação com metodologia mais próxima da que utilizamos avaliou as mudanças da função cardíaca em participantes de uma trilha ecológica e constatou a relação das ondas mitral E/A e Doppler tecidual E' significativamente diminuídas, a partir de 21 km.^37^

O treinamento regular e aeróbio pode minimizar as alterações agudas da função diastólica frente a solicitação de maiores demandas cardíacas no exercício intenso. Este efeito de treinamento pode exercer um papel fundamental na preservação do preenchimento diastólico em atletas mais velhos.^35^^,^^38^

Encontramos correlações positivas entre os seguintes: E/A e E'; CK-MB (massa) e troponina T; e CK-MB (massa) e NT-proBNP. Correlação negativa foi encontrada apenas entre CK-MB (massa) e E/A (relação da velocidade de enchimento ventricular rápido com a velocidade de contração atrial).

Poucos trabalhos realizaram correlação das ondas da função diastólica e das variações séricas dos biomarcadores de lesão cardíaca com as ondas de variação da função diastólica. Sabe-se que em padrões fisiológicos as variações do comportamento do fluxo mitral refletem na mesma direção que as variações do comportamento tecidual ventricular.^13^ Jouffroy et al.^37^ conseguiram encontrar correlação positiva entre E/A e E' em participantes amadores de prova de resistência. Os poucos estudos que avaliaram essas correlações têm resultados discordantes.^37^

Sabe-se da relação direta do aumento dos BNP(s) com as troponinas, creatina quinases e com as alterações diastólicas. A troponina T também está fortemente associada às anormalidades de relaxamento ventricular.^16^^,^^20^^,^^39^ A correlação positiva da CK-MB (massa) com troponina T e NT-proBNP encontrada em nosso trabalho pode estar relacionada ao fato de que mesmo em situações de ausência de isquêmia cardíaca, níveis mínimos desses biomarcadores podem ser liberados na corrente sanguínea e na mesma direção do comportamento da CK-MB (massa).

Por conseguinte, verificamos correlação inversa da CK-MB (massa) com a relação das ondas E/A. O motivo que a diminuição de um implica no aumento da outra necessita ser mais estudado já que, talvez, essa correlação possa ser apenas acaso. Podemos especular quanto à possibilidade de que a disfunção diastólica reversível seja um dos possíveis mecanismos de aumento plasmático da CK-MB (massa) com a diminuição da onda E e aumento da onda A.

### Limitações

A coleta de dados durante os dias do trajeto teve que se adaptar aos horários disponíveis do evento, podendo ter interferido na avaliação das variações agudas dos biomarcadores cardíacos já que os níveis plasmáticos destes variam conforme o tempo de exposição. Não foi possível, também, realizar coleta matinal para avaliar o comportamento das variáveis após o repouso, já que isso atrapalharia o descanso e o processo de recuperação dos participantes que tinham que acordar as quatro horas da manhã para se preparem e depois percorrerem os trechos estabelecidos. Além disso, a avaliação pós exposição poderia ter ajudado a elucidar melhor questões referentes ao comportamento dos biomarcadores e das ondas da função diastólica como, por exemplo, se houve ou não retorno próximo aos valores basais.

## Conclusões

Os efeitos da atividade física intensa, prolongada e intercalada foram verificados a partir das variações significativas no comportamento da CK-MB (massa), NT-proBNP e E'. Vale ressaltar que apesar das alterações encontradas, não houveram critérios demonstrativos de dano ao miocárdio com essa modalidade de esforço em indivíduos treinados.
