# Supplementary material for: Função Diastólica e Biomarcadores de Participantes de Caminhada de Longa Distância
Source: Arq Bras Cardiol. 2020 Oct 13;115(4):620–7. [Article in Portuguese] doi: 10.36660/abc.20190271 (PMC8386974; doi:10.36660/abc.20190271)
Supplement: Translation

# Translation: Diastolic Function and Biomarkers of Long-Distance Walking Participants

**Authors:** Maicon Borges Euzebio, Priscila Valverde de O. Vitorino, Watila Moura Sousa, Milena Andrade Melo, Sérgio Henrique Nascente Costa, Ana Luiza Lima Sousa, Thiago de Souza Veiga Jardim, Ana Carolina Arantes, Paulo Cesar B. Veiga Jardim, Weimar Kunz Sebba Barroso

**Affiliations:** 1 GoiâniaGO Brazil Universidade Federal de Goiás – Medicina, Goiânia, GO – Brazil; 2 GoiâniaGO Brazil Pontifícia Universidade Católica de Goiás, Goiânia, GO – Brazil; 3 GoiâniaGO Brazil Universidade Federal de Goiás – Pós-graduação em Ciências da Saúde, Goiânia, GO – Brazil; 4 GoiâniaGO Brazil Universidade Federal de Goiás – Liga de Hipertensão Arterial, Goiânia, GO – Brazil; 5 GoiâniaGO Brazil Universidade Federal de Goiás – Cardiologia, Goiânia, GO – Brazil; 6 GoiâniaGO Brazil Faculdade da Polícia Militar do Estado de Goiás, Goiânia, GO - Brazil

**Keywords:** Walking, Biomarkers, Biological, Blood Pressure, Troponin-T, Natriuretic Peptide Brain, Athletes, Echocardiography, Doppler/methods

## Abstract

**Background:**

The effects of long-distance walking on the cardiovascular system have been little studied.

**Objectives:**

The general objective of this study was to verify these effects on the behavior of diastolic function and the cardiac biomarkers CK-MB (mass), troponin T, and NT-proBNP, in amateur athletes.

**Method:**

This longitudinal study, conducted in 2015, evaluated participants during the following 5 stages: E0 (baseline) before starting the trajectory and the others, E1, E2, E3, and E4, at the end of each day, totaling 244.7 km. At all stages, the biomarkers NT-proBNP, CK-MB (mass), and troponin T were measured. Echocardiogram was performed to analyze the E, A and E' waves. P < 0.05 was adopted as significant.

**Results:**

The study evaluated 25 participants, with an average age of 46 ± 10.5 years and body mass index of 20.2 ± 2.3 kg/m2. Increased values were found for NT-proBNP from E0 to E1, E2, E3, and E4 (p < 0.001), CK-MB (mass) from E0 to E2 (p < 0.001), and E' wave from E0 to E1, E2, E3, and E4 (p < 0.001). Positive correlations were identified between the following: CK-MB (mass) and troponin T (E1: r = 0.524, p = 0.010; E4: r = 0.413, p = 0.044); CK-MB (mass) and NT-proBNP (E4: r = 0.539, p = 0.006); and E/A and E' (E0: r = 0.603, p < 0.001; E1: r = 0.639, p < 0.001; E4: r = 0.593, p = 0.002). A negative correlation was found between CK-MB (mass) and E/A (E1: r = −0.428, p = 0.041).

**Conclusion:**

The effects of intense, prolonged, and interspersed physical activity were verified based on significant variations in the behavior of CK-MB (mass), NT-proBNP, and the E' wave. Notwithstanding the alterations found, there were no criteria suggestive of myocardial damage

## Introduction

Physical exercise is fundamental to general health maintenance and disease prevention.^1^ Intensity, duration, and frequency, however, are factors that separate benefit from harm in the human organism. Recent evidence has asked whether the physiological demand of maintaining elevated cardiac output during a prolonged period of exercise may result in transient impairment of cardiac functions.^2^

The acute effects of physical exercise on diastolic function and even variations in diastolic dysfunction are indirectly related to persistent stimulation, which also depends on intensity and duration. These adaptations, even when they are short-term, may cause decreased cardiac function, which is known as cardiac fatigue.^3^

The cardiac enzymes creatine kinase-MB (CK-MB) and cardiac troponin T (cTnT), and the amino acid precursor N-terminal pro B-type natriuretic peptide (NT-proBNP) are important biomarkers for evaluating the existence of myocardial lesions and diastolic function.^4^

CK-MB levels may also increase in states of rhabdomyolysis and even apoplexy; in this manner, they have variable sensitivity.^5^ Troponin T is the preferred marker of myocardial lesion, and it is considered the gold standard.^6^

NT-proBNP is associated, in a direct and parallel manner, with brain natriuretic peptide (BNP) concentrations. It may be used for diagnostic and prognostic assessment of left ventricular insufficiency, and it also increases in conditions that induce diastolic dysfunction. Little is known regarding the behavior of these biomarkers and especially their prognostic meaning in healthy individuals undergoing intense physical stress.^7^

Nonetheless, few studies have evaluated the effect of moderate to high intensity long-distance walking on the cardiovascular system based on evaluation of diastolic function and cardiac biomarkers. This was the first study to evaluate diastolic function and cardiac biomarkers in this type of exercise. Accordingly, the objective of this study was to verify, in amateur athletes, the effects of moderate to high intensity long-distance walking on the behavior of diastolic function and the cardiac biomarkers CK-MB (mass), troponin T, and NT-proBNP.

## Methods

This was a longitudinal study, carried out during a long-distance walk, namely, the Goiás Ecological Walk, in the year 2015. Initially, approximately 200 people signed up via Internet to participate. The candidates participated in a selection process, during which they walked 56 km, divided into two days (28 km each), which had to completed in, at most, three hours and ten minutes for men and three and a half hours for women. Participants were ranked according to the best times in each age group, and a total of 29 participants, 25 male and 4 female, were selected. Women were not included in analysis, because they did not complete the previously established daily route.

The research project received approval from the Research Ethics Committee of the Pontifical Catholic University of Goiás under certificate number 1.107.021. The individuals approved during the selection process were invited to participate in the study. Following acceptance, a free and informed consent form was applied, and initial evaluation (E0) was carried out in a cardiovascular evaluation unit. Other evaluations took place 34 days after E0 during the 244.7-km trajectory, at the end of each day, at the resting places in each city on the trajectory, from July 21 to 24, 2015; evaluation days were named E1, E2, E3, and E4 (first, second, third, and fourth days of evaluation). Data collection was not carried out on the final day of the event, as participants were released to return to their home cities.

The predominant relief of the terrain was described as ascending, descending, and level, according to each day of the trajectory. This information was obtained from a previous study that had evaluated this.^8^ The slope of the terrain was obtained using ArcGIS 10.3 software, through the slope tool (slope, arctoolbox), based on Shuttle Radar Topography Mission (SRTM) images provided by the American geological service, with pixel size of three arcsec. For meteorological monitoring of the trajectory days (E1 to E4), data were utilized from the Goiás station officially denominated by the Brazilian National Institute of Meteorology (INMET, acronym in Portuguese) of E014.

### Organization of Collection

The study setting was subdivided into stations, one for each evaluation. Evaluations consisted of anamnesis (during E0 only) and echocardiography and blood collection for evaluation of biomarkers at all stages. Collection of echocardiographic data and biomarkers began around 6:00 p.m. (30 to 120 minutes after the end of each walk) on every day of the trajectory. In this manner, when participants arrived at the resting places, research stations had already been prepared, initiating the flow of identification of participants, followed by blood collection and echocardiographic evaluation; participants were subsequently free to return to the event. Collection was not possible in the morning, as participants woke up at 4:00 a.m., and this could have interfering with their periods of rest and recovery.

The researchers organized anamnesis in the form of an interview with questions regarding personal data such as age (years), sex (male and female), profession, and marital status; personal and family history of arterial hypertension, diabetes, and dyslipidemia; use of medication(s); tobacco use (current/prior); number of cigarettes per day, for smokers; physical exercise practice (weekly frequency, daily duration, and duration of practice); and the presence of orthopedic problems that might make it difficult to practice exercise.

### Blood Biomarker Tests and Echocardiogram Procedures

The blood collection technique was via peripheral venous access in left cubital fossa.^9^ Approximately 5 ml of blood were drawn from each participant. The blood was centrifuged at the collection site at 3,000 rpm for 10 minutes to obtain serum/plasma. The serum was collected in sample tubes with separator gel. The samples were promptly stored and frozen at −20 ºC in a specific freezer at the collection stations.^10^ The samples were sent to an accredited laboratory for analysis. Kits registered by the Brazilian National Health Surveillance Agency (ANVISA, acronym in Portuguese) were used for the biomarker tests. The values of CK-MB (mass), troponin T, and NT-proBNP were obtained using the electrochemiluminescence immunoassay technique.^11^ The assays were performed on a COBAS® Modular Analytics E170 system using the following kits: CK-MB STAT, Troponin T hs, and proBNP II, respectively. To ensure that assays were performed correctly, all instructions provided in the user's guide for analyzers were followed. A biomedical doctor handled all exams. The following units of measurement were used: pg/ml for NT-proBNP and troponin T and ng/ml for CK-MB (mass).^11^

### Echocardiography

Echocardiogram was carried out individually at all stages of collection, during the day on E0 and around 6:00 p.m. from E1 to E4, after participants' arrival at the research stations. All exams were carried out by the same echocardiographer. One- and two-dimensional echocardiogram with color Doppler was performed using a Philips CX50® portable device, with an electronic transducer with 2 to 5MHz of frequency. The technique and reference standards used were those recommended by the American Society of Echocardiography ^(12)^. The same angle of incidence was used for transmittal analysis, attempting to align the ultrasound beam as parallel as possible to the color Doppler flow. The parameters used for analysis of diastolic dysfunction, with their respective units of measurement, were, for mitral flow, E in cm/s, A in cm/s, and E/A ratio. For tissue evaluation, E' in cm/s was used.^13^

### Statistical Analysis

Data were analyzed using descriptive statistics with absolute and relative frequencies, averages, standard deviation, and confidence interval. The Shapiro-Wilk test was used to test the normality of the data distribution of the variables. To compare variables related to cardiac biomarkers between the days of the walk and echocardiographic parameters, the ANOVA test for repeated measures was applied, followed by Bonferroni's post hoc test. Pearson or Spearman tests were used for correlations. P value < 0.05 was adopted for statistical significance. Stata software, version 14, was used to carry out statistical analysis.

## Results

### Characteristics of the Sample

Evaluation included 25 men, with average age of 46 ± 10.5 years and body mass index 20.2 ± 2.3 kg/m^2^ (Table 1). Four (16%) participants were using medications that did not influence the variables analyzed. The distance covered in four days was 244.7 km, with an average velocity of 7.6 km/h. All participants walked together as a group, thus maintaining the same velocity. The nocturnal resting period was between six and seven hours daily.

**Table 1:** Social, health, and lifestyle characteristics of the participants of the Goiás Ecological Walk, Brazil, 2015 (n = 25)*

Variables	n	%
**Age range (years)**		
19 – 39	5	20
40 – 59	19	76
≥ 60	1	4
**Medication use**		
No	21	84
Yes	4	16
**Diseases reported**		
Diabetes mellitus	1	4
Dyslipidemia	1	4
Hypothyroidism	2	4
Orthopedic problems	3	12

* Number of participants evaluated.

In relation to previous physical exercise, 24 participants (96%) practiced running; one (4%) practiced cycling, and four (16%) practiced bodybuilding. Average duration of aerobic activity was 13.3 years (0.5 ± 40), and average weekly distance of running, which was the most reported activity, was 64 km.

### Environmental Characteristics of the Trajectory

Temperature during the days of the walk ranged from 19 to 32 ºC, and relative humidity ranged from 21% to 77%. On E1 and E3, the relief was predominantly ascending; on E2, it was descending (E2), and, on E4, it was level. The most accentuated variations in the slope of the terrain were found in sections of E2 and E3, ranging from 0 to −15.

### Evaluation of Diastolic Function Biomarkers and Waves During the Long-Distance Walk

NT-proBNP showed a significant increase from E0 to all the other evaluations. CK-MB (mass) showed a significant increase from E0 to E2, and troponin T did not show any significant alterations. Regarding diastolic function, the E and A waves did not alter between the days of the walk. The E' wave increased from E0 to all the other days (Figure 1).

**Figure 1:** Comparison between NT-proBNP, troponin T, CK-MB (mass), E/A, E, and E' values obtained during baseline evaluation and the four days of exposure to moderate to intense physical activity.

Correlations were made between all parameters collected regarding diastolic function biomarkers and waves; only significant correlations are shown. In relation to blood biomarkers, positive and moderate correlations were identified on the following days of exposure: E1, between troponin T and CK-MB (mass); and E4, between NT-proBNP and CK-MB (mass), as well as between troponin T and CK-MB (mass). In relation to diastolic function, positive and moderate correlations were identified between E/A and E' on E0, E1, and E4. With respect to diastolic function biomarkers and waves, a negative and moderate correlation was found between CK-MB and E/A on E1 (Table 2).

**Table 2:** Correlation of biomarkers, diastolic function, and biomarkers with diastolic function in participants of the Goiás Ecological Walk, Brazil, 2015 (n = 25)

Evaluation	Parameter	r	p
E0	E/A x E’	0.603	0.001*
			
	Troponin T x CK-MB (mass)	0.524	0.010**
E1	E/A x E’	0.639	0.001*
	CK-MB (mass) x E/A	-0.428	0.041**
			
	NT-proBNP x CK-MB (mass)	0.539	0.006**
E4	Troponin T x CK-MB (mass)	0.413	0.044**
	E/A x E’	0.593	0.001*

* Pearson test;

** Spearman test.

## Discussion

Although the participants were considered amateur athletes, the majority had prior experience with the trajectory and in prior editions of the Goiás Ecological Walk, with an average of 7.2 participations; in addition to this, the selection process resulted in a highly select sample.

The velocity of competitive running in half marathons or marathons solicits higher energetic and metabolic demands, and it increases the chances of cardiovascular outcomes, especially in individuals who are not conditioned.^14^ In our study, the average velocity was 7.6 km/h, which is much lower than in marathons and half marathons; for this reason, there were lower energetic and metabolic demands on participants.

During the days they were exposed to exercise, in comparison to the 30 preceding days (E0), significant increases occurred in NT-proBNP, CK-MB (mass), and E' wave (tissue Doppler) values. We did not observe significant variations in the levels of troponin T and the mitral flow E/A ratio.

This increase has also been found in healthy individuals, adolescents, and adults, regardless of sex, when undergoing resistance exercises, with a return to baseline values during the first 24 hours after exercise.^15^^,^^16^

In the present study, we found an up to five-fold increase in serum NT-proBNP concentrations following exercise, when compared to baseline levels; these data corroborate the literature.^17^^–^^19^ They also reinforce the importance of the natriuretic effect in the mechanism of acute and subacute adaptation of the cardiovascular apparatus to physical effort.

Serum elevations of CK-MB (mass) have also been found in individuals after strenuous exercise. In a survey conducted in the United States, with young participants in a marathon, increased and sustained plasma levels of CK-MB were found up to 54 hours after exposure to exercise.^20^^,^^21^

Trained individuals, who perform high intensity exercise in sports, during the maximal effort test, tend to present acutely higher serum elevations of CK-MB than untrained individuals, thus suggesting cardiac involvement that is mainly benign in the nature of these serum levels.^22^

Scientists have also verified the effect of prolonged exercise and variations in environmental temperature on the behavior of creatine kinases, and they identified that the longer the exercise and the higher the environmental temperature, the greater the plasma release of these biomarkers.^23^ The longer the stretch covered in a shorter amount of time, the greater the chances of muscle damage and, thus, the greater serum quantity of creatine kinases. Another important fact is that, during descent, the ground reaction force exerted against participants may also lead to increased musculoskeletal injuries, due to the greater source of impact, with a tendency toward more accentuated metabolic cost during descending slopes.^24^^,^^25^

In our study, significant variations in CK-MB (mass) were found only between E0 and E2. This increase may have been related to the characteristics of the trajectory in E2, namely, predominantly descending, with greater variation in slope and greater distance than the other days. The cumulative effect of CK-MB (mass) in E1 may have influenced the significant increase in the value of CK-MB (mass) in E2.

Evidence also exists regarding the limitations of evaluation of CK-MB in healthy individuals during physical exercise practice. Its specificity becomes impaired in the presence of inflammatory processes and skeletal muscle stress associated with a reduction during the first hours of exposure to effort, due to the slowed appearance of these markers in the blood, and it is more sensitive when cardiac injury is present.^26^

In studies on half marathons, marathons, and 48-hour ultra-marathons, carried out in amateur runners (non-athletes), elevated troponin T values were found during the first three hours of post-exposure, with important reductions immediately following, reaching baseline levels in a maximum of 48 hours.^20^^,^^27^ In swimmers, 60 minutes after swimming, the same variation was found in troponin T as in amateur runners.^16^

Eijsvogels et al.^28^ evaluated 82 people during a trajectory of 30 km daily for four days, and they also found that troponins increased only on the first day, with plasma reduction on all other days showing an association with walking speed.^28^ A meta-analysis of 45 studies that evaluated the behavior of troponins and BNPs following exposure to resistance exercises observed that the elevated plasma values of troponins and BNP during and after intense and prolonged exercise are prone to change, and they may represent an acute characteristic with respect to exposure to exercise.^29^ The biomechanics of the release of troponins induced by physical exercise are still unclear; it is also unclear whether this really reflects a physiological or pathological process.^30^

In this study, troponin T did not show a significant increase during the days of the trajectory; these results differ from those found in other studies mentioned. This may be related to following facts: the participants covered the trajectories at an average speed below competition values, even for amateur runners; they alternated running and walking; and they were carefully hydrated throughout the journey. The behavior of troponin T was different than what is generally expected when related to CK-MB (mass). As troponin T has greater specificity for myocardial damage due to ischemia, it is possible that this is the reason for the lower increase between E0 and the other days. This fact may demonstrate that damage to the cardiovascular system seems to be minimal and this exercise modality seems to be safe.^31^

In our sample, we found a significant increase in the E' wave in relation to baseline values, without a reduction in the E/A wave ratio. These findings may be related to the characteristics of the population evaluated, which was well conditioned, and to the intensity of the effort, contributing to a greater adaptive capacity of myocardial remodeling. Other studies that have performed echocardiography in different populations have found different results. Left or right ventricular diastolic dysfunction was not found in amateur medium- and long-distance triathletes.^32^ In contrast, in adult athletes, alterations in myocardial relaxation were demonstrated during diastole,^33^ identified by increased E wave (mitral flow) in comparison with the E' wave (tissue Doppler). In ultra-resistance exercises, there were significant decreases in the E wave and the E/A ratio immediately after exercise.^34^^–^^36^ A study, whose methodology was closer to the one we used, evaluated changes in cardiac function in participants of an ecological walk and found significantly decreased mitral E/A ratio and tissue Doppler E' wave, from 21 km.^37^

Regular aerobic training can minimize acute changes in diastolic function in response to the greater cardiac demand during intense exercise. This training effect can play a fundamental role in preserving diastolic filling in older athletes.^35^^,^^38^

We found positive correlations between the following: E/A and E'; CK-MB (mass) and troponin T; and CK-MB (mass) and NT-proBNP. A negative correlation was found only between CK-MB (mass) and E/A (the ratio of the velocities of rapid ventricular filling and atrial contraction).

Few studies have made correlations of diastolic function waves and serum variations of cardiac injury biomarkers with diastolic function variation waves. It is known that, in physiological patterns, variations in the behavior of mitral flow are reflected in the same direction as variations in ventricular tissue behavior.^13^ Jouffroy et al.^37^ succeeded in finding a positive correlation between E/A and E' in amateur participants of ultradistance running. The few studies that have evaluated these correlations have found discordant results.^37^

The direct relation of increased BNP(s) with troponins, creatine kinases, and diastolic alterations is known. Troponin T is also strongly associated with ventricular relaxation abnormalities.^16^^,^^20^^,^^39^ The positive correlation of CK-MB (mass) with troponin T and NT-proBNP found in our study may be related to the fact that, even in situations where cardiac ischemia is absent, minimum levels of these biomarkers may be released into the bloodstream, in the same direction as the behavior of CK-MB (mass).

Consequently, we found an inverse correlation of CK-MB (mass) with the E/A wave ratio. The reason that the decrease in one leads to an increase in the other needs to be studied further, given that this correlation may be merely casual. We can speculate as to the possibility that reversible diastolic dysfunction is one of the possible mechanisms of plasma increase of CK-MB (mass) with the decreased E wave and the increased A wave.

### Limitations

Data collection during the days of the trajectory had to adapt to the available times of the event, and this may have interfered with evaluation of acute variations in cardiac biomarkers, given that plasma levels vary according to time of exposure. It was also not possible to carry out collection in the morning in order to evaluate the behavior of the variables after resting, as this would have interfered with participants' rest and the recovery process, as they had to wake up at 4:00 a.m. to get ready and then walk the established route. Furthermore, evaluation of exposure would have helped better elucidate questions regarding the behavior of diastolic function biomarkers and waves, for instance, whether or not there was a return close to baseline values.

## Conclusions

The effects of intense, prolonged, and interspersed physical activity were verified based on significant variations in the behavior of CK-MB (mass), NT-proBNP, and E'. It is worth underscoring that, notwithstanding the alterations found, there were no criteria demonstrative of myocardial damage during this exercise modality in trained individuals.
